# Fine Mapping and Candidate Gene Analysis of the Tiller Suppression Gene *ts1* in Rice

**DOI:** 10.1371/journal.pone.0170574

**Published:** 2017-01-20

**Authors:** Lei Liu, Fen Meng, Yonggang He, Menghao Zhu, Yanhao Shen, Zhihong Zhang

**Affiliations:** State Key Laboratory of Hybrid Rice, College of Life Sciences, Wuhan University, Wuhan, China; Clemson University, UNITED STATES

## Abstract

Tiller number is one of the key factors that influences rice plant type and yield components. In this study, an EMS-induced rice tiller suppression mutant *ts1* was characterized. Morphological and histological observations revealed that, in the *ts1* plants, the tiller buds were abnormally formed and therefore cannot outgrow into tillers. With an F2 population derived from a cross between *ts1* and an *indica* cultivar Wushansimiao, a major gene, *tiller suppression 1* (*ts1*) was fine-mapped to a 108.5 kb genomic region between markers ID8378 and SSR6884 on the short arm of rice chromosome 2. Candidate gene analysis identified nineteen putative genes. Among them, *ORF4* (LOC_Os02g01610) is a PPR gene which harbored a point mutation c.+733/C→T in *ts1* mutant plants. A co-dominant SNP marker cd-733C/T was subsequently developed and the SNP assay demonstrated that the point mutation co-segregated with tiller suppression phenotype. Quantitative RT-PCR analysis showed that the expression level of *ORF4* in *ts1* plants was significantly lower than that in their wild plants, and the expression of rice tillering regulators *MOC1* and *HTD1* was also significantly decreased in *ts1* plants. Our data indicated that *ORF4* was a strong candidate gene for *ts1* and *ts1* might play a role in regulating rice tillering through *MOC1* and *HTD1* associated pathway. The results above provide a basis for further functional characterization of *ts1* and will shed light on molecular mechanism of rice tillering. The informative SNP marker cd-733C/T will facilitate marker-assisted selection of *ts1* in rice plant type breeding.

## Introduction

Rice is one of the most important cereal crops for more than half of the world’s population [1]. Tiller number is considered to be one of the key factors that affects rice plant architecture and grain yield because of its involvement in determining the final number of productive panicles [2]. For a long time, desirable plant architecture which focused on tiller number has been the main goal for breeders to breed rice varieties with high-yield potential. A famous example was the “New Plant Type” rice varieties with low tiller number and large panicles, which were developed by the International Rice Research Institute [3]. For a given rice variety, its tillering ability is mainly determined by its genetic background although it could be affected by environmental conditions such as light, temperature, plant density, nutrient and water supply [4]. Therefore, identifying and mining genetic factors that contribute to rice tiller number variation are crucial for rice plant architecture improvement.

To enlarge rice tiller-regulating gene pools, scientists made great efforts by identifying novel rice tillering mutants generated from different mutagens and dissecting their corresponding genetic factors. Thus far, three monoculm genes have been identified and mapped onto different rice chromosomes. Among them, *MOC1*, a member of the *GRAS* family in rice, controls the formation of axillary buds and promotes their growth [5]. *MOC2*, encodes cytosolic fructose-1, 6-bisphosphatase 1 (FBP1), is essential for the outgrowth of tiller buds in rice [6]. *MOC3*, in which a point mutation causes the premature termination of *OsWUS*, is required for the formation of axillary buds of rice [7]. During the past decade, a series of EMS-induced *reduced culm number* (*rcn*) mutants and their responsible genes have been characterized in rice. Among those genes, *RCN8* and *RCN9* have been mapped on the short arm of chromosome 6 and the long arm of chromosome 1 respectively [8]. *RCN1*, mapped on the short arm of chromosome 3, encodes an ATP-binding cassette subfamily G protein which is required for rice shoot branching [9]. A semi-dominant quantitative trait locus, *OsSPL14*/*IPA1*/*WFP*, was genetically mapped on the long arm of chromosome 8 and it was found to be regulated by miRNA156 and work as a transcription factor to shape an ideal plant type [10, 11].

Besides agronomic importance, studies on rice tillering could also contribute to a better understanding toward plants branching. As a monocot model species, rice may share conserved branching regulation mechanisms with other species, such as *Arabidopsis* and maize [2]. For instance, rice branching regulators *D3*, *D17*/*HTD1/DIT*, and *D10* have been characterized as *MAX2*, *MAX3*, *MAX4* homologs in *Arabidopsis*, respectively [12–15]. *TAB1/OsWUS*, which was reported to encode an ortholog of *Arabidropsis WUS*, is involved in regulating rice axillary meristem formation [16]. In rice, *OsTB1*/*FINE CULM1* was characterized by the homologous clone strategy from maize *TB1* gene. *OsTB1* encodes a TCP domain transcription factor, acting downstream of strigolactone pathway as an integrator of multiple signaling pathways in shoot branching of rice [17, 18].

Considering the great importance in yield improvement and biological value of rice tillering, it is imperative to identify novel mutants with altered tiller numbers and mine their corresponding alleles. The present study was undertaken to characterize a novel EMS-induced tiller suppression mutant *ts1* in rice and fine map the major gene *ts1*. The candidate genes for *ts1* were analyzed.

## Materials and Methods

### Plant materials

The rice mutant *ts1* was isolated from an EMS-mutagenized M4 line and its wild type is LY95, a *japonica* cultivar. The mutant plant was then self-crossed for five generations to ensure its genetic homozygosity. A Chinese *indica* cultivar Wushansimiao was used as male parent to cross with the mutant *ts1* to develop F1 plants. A large F2 population of 9700 individuals was obtained from these self-pollinating F1 plants for further linkage analysis and molecular mapping. In the summer of 2015, the F1 and F2 plants together with their both parents were planted at Ezhou Experimental Station of Wuhan University (Hubei Province, China, 30°23′N, 114°52′E). The planted density was 16.7 cm between plants within a row and 20.0 cm between rows. Filed management followed the standard agricultural practice.

For LY95, the *ts1* mutant, and Wushansimiao, ten representative plants in the middle of each accession plot were sampled to count their culm number (one main culm together with its tiller culms for each rice plant) at 20–70 DAS and their reproductive culm (culm with 10 or more grains) number at maturity. Plant height was measured from the ground to the tallest panicle tip of a plant at maturity. Panicle length was measured as the length from the panicle neck to panicle tip of the main culm panicle. Spikelets of a main culm panicle were calculated as the sum of filled and unfilled spikelets harvested from the main culm panicle of a plant. For each trait, average trait value of 10 representative individuals was calculated. The independent sample *t*-test program and Duncan’s test program of SPSS 10.0 for Windows were conducted to detect significances between mean values.

### Histological analysis of tiller buds

For the mutant *ts1* and its wild type LY95, their basal part of rice main culms (about 1 cm) with tiller buds was sampled from 30-day-old seedlings at five leaf stage. The samples were then fixed in formalin/acetic acid/alcohol (FAA), followed by dehydration through a graded ethanol series and embedding in Paraplast Plus. Sections were stained with 1% toluidine blue and observed under bright-field microscopy (IX71, Olympus). Micrographs were taken with Image-Pro Insight software (Media Cybernetics, America).

### Molecular mapping of target gene

The bulked segregant analysis (BSA) method was employed to identify molecular markers genetically linked to target gene [19]. A pair of DNA bulks were constructed by pooling equal amount of DNA from 10 F2 plants which have extremely low (culm numbers = 1 or 2) and high (culm numbers≥12) tillering phenotypes to construct a low-tillering bulk (L bulk) and a high-tillering bulk (H bulk), respectively. The two DNA bulks along with the DNA samples of two parents were used as templates for BSA. A total of 269 polymorphic SSR markers distributed over all of 12 rice chromosomes were used to screen markers putatively linked to the mutant trait.

For local linkage map construction, 279 *ts1*/Wushansimiao F2 individuals were randomly sampled from the planted plot (except the edge ones of each row). Genomic DNA was extracted from fresh leaf of each individual by the CTAB method [20] and used for marker genotyping. For the putatively linked markers identified in BSA analysis on the short arm of rice chromosome 2, the genotype data of the 279 F2 plants was used to construct a local linkage map by Mapmaker/Exp 3.0 [21]. The Kosambi function was used to calculate genetic distance between markers.

For QTL mapping, five associated phenotype traits including culm number, reproductive culm number, plant height, panicle length, and spikelets of main panicle were investigated for each of the 279 F2 individuals. Culm number was investigated three times at 40, 50, 60 DAS, respectively. The other traits were measured at maturity as described above for the parents. With the trait phenotype data and marker genotype data of the 279 F2 individuals, QTL analysis was performed by QTLMapper 1.0 based on mixed linear models [22]. Composite interval mapping was conducted to locate and validate the main-effect QTLs. The threshold for putative QTL validation was a LOD score of 3.0. Genetic parameters associated with the significant main-effect QTLs, such as QTL marker intervals, LOD values, phenotypic variance component explained by each QTL (R^2^), were estimated at the positions of respective maximum LOD peak in each putative QTL region.

The recessive-class analysis (RCA) was then conducted to fine map the target gene. A fine mapping population of 497 F2 individuals with extremely low tillering phenotypes (culm number = 1 or 2) were selected out from a large *ts1*/Wushansimiao F2 population with 9700 individuals. For all of the F2 individuals, their recessive phenotypes were further verified by their F3 progenies in the next season. Based on the QTL mapping results, two putatively linked markers RM12298 and RM154 were identified to flank the *ts1* locus. Then, according to the physical map of the rice cultivar Nipponbare, seven polymorphic SSR and InDel markers were identified to distribute across the interval RM12298-RM154 on the short arm of rice chromosome 2. Finally, the seven polymorphic markers together with flanking markers RM12998 and RM154 were used to detect recombinant events from 497 recessive F2 individuals. As a result, the target gene *ts1* was fine mapped to a narrow marker interval.

### Source of marker primers and PCR amplification

The primer sequences of most of SSR markers used in this study were downloaded from the Gramene SSR Markers Resource (http://archive.gramene.org/markers/microsat/). A part of SSR markers such as SSR6884 was developed as below. We downloaded the genomic sequence of Nipponbare between markers RM3340 and RM12329 from NCBI (http://www.ncbi.nlm.nih.gov), and put the sequence into the SSR Hunter tool (http://www.bio-soft.net/dna/SSRHunter.html) to find simple sequence repeats. For the founded simple sequence repeats, their ~500 bp flanking genomic sequence was used to design SSR primers (S1 Table) by Primer Premier 5.0 (PREMIER Biosoft International, Palo Alto, California, USA). To develop Indel markers such as ID8378, *japonica* cv. Nipponbare’s genomic sequence between markers RM12317 and RM3340 was downloaded from the NCBI database (http://www.ncbi.nlm.nih.gov) and the corresponding *indica* cv. 9311’s sequence was also downloaded from RIS^*e*^ database (http://rise2.genomics.org.cn/page/rice/index.jsp). The two cultivars’ sequences were compared by DNAMAN software (Lynnon Biosoft, USA) to find insertion and deletion. For the founded insertion and deletion, their flanking sequences were used to design InDel primers by Primer Premier 5.0.

PCR amplifications for these SSR and Indel markers were conducted at 94°C for 5 min, followed by 35 cycles of 94°C for 30 s, 55°C for 30 s, 72°C for 1 min, ended with an extension step of 72°C for 7 min. PCR products were separated by 6% denatured polyacrylamide gel electrophoresis and then visualized by silver staining.

### Estimation of dominance degree

Dominance degree (*H*) for the studied trait or the target gene was calculated as below:

$$H=\frac{F1-\left(P1+P2\right)/2}{\left(P1-P2\right)/2}$$ (1)

$$H=\frac{TW-\left(TT+WW\right)/2}{\left(1-2r\right)\left(TT-WW\right)/2}$$ (2)

Formula (1) given by Falconer D. S. [23] was used to calculate dominance degree of the trait culm number of the *ts1* mutant relative to the wild type Wushansimiao. *P1*, *P2*, *F1* were trait values of the two parents *ts1*, Wushansimiao and their F1 plants, respectively. Formula (2) given by Edwards M. D. et al [24] was used to calculate dominance degree of the target gene *ts1* relative to its wild type gene. For a marker putatively linked to the target gene, *TT*, *WW*, *TW* represents trait values of mutant type, wild type and heterozygous genotypes in a segregating population, respectively. *r* represents recombination frequency between a putatively linked marker and the target locus. Here, *H* = -1 would be indicative of complete recessivity, -1 < *H* < 0 indicate incomplete recessivity and *H* = 0 suggests no dominance or recessivity.

### Cloning and sequencing of the candidate genes

According to the fine mapping results and with Nipponbare’s genomic sequence as a reference, we obtained the candidate ORFs from the Rice Genome Annotation Project Database (http://rice.plantbiology.msu.edu/index.shtml). For each of the ORFs, its flanking genomic sequence was downloaded from NCBI database and then was put into Primer Premier 5.0 as template to design cloning primers (S2 Table). High fidelity LA-Taq (TakaRa, Dalian, China) was used for amplification of the candidate genes (TakaRa, Dalian, China). PCR products were sub-cloned into pMD18-T cloning vector according to the manufacturer’s specification and then sequenced at the Quintara Biosciences Co., Ltd (Wuhan). Sequence contigs were assembled and aligned using the Sequencher Program (Gene Codes Corporation, Ann Arbor, MI).

### Gene expression analysis

To develop gene expression primers, cDNA sequence of each gene was obtained from the Sequence Display interface of Rice Genome Annotation Project Database (http://rice.plantbiology.msu.edu/cgi-bin/gbrowse/rice/) and then the cDNA sequence was put into Primer Premier 5.0 as template to design its expression primers. The expression primers of five genes (*ORF4*, *OsSPL14*, Os*TB1*, *MOC1*, *HTD1*, and *D3*) were listed in S3 Table.

For quantitative RT-PCR, total RNA was isolated with TRIzol (Invitrogen) reagent from the shoot apexes of rice seedlings at tillering stage (40 DAS). RNA sample (1 μg) was treated with DNaseI and then used for cDNA synthesis with the Super-Script III first-strand cDNA synthesis system (Invitrogen) according to the manufacturer’s instructions. cDNA template was amplified using 2×SYBR Green PCR Master Mix (Takara) on ABI 7500 (Applied Biosystems) machine. PCR reactions were performed in triplicate for each sample. The relative expression level of each transcript was obtained by normalizing the *OsUBI* signal based on the 2 –^ΔΔCT^ method. Amount of the transcripts in the wild-type plant was set at 1.0.

### Development of a codominant SNP marker

Based on the single nucleotide polymorphism (SNP) between wild type and *ts1* in LOC_Os2g01610, a co-dominant SNP marker was developed by following the tetra-primer ARMS-PCR method [25]. At the 3’-terminal base position, primers CR and TR were complementary to the mutant and the wild type alleles, respectively. To enhance allelic specificity, an artificial base pair mismatch was designed at the 3rd base pair from the 3’-end of the reverse primers CR and TR, respectively (S3 Table). The PCR procedure for marker was conducted at 94°C for 5 min, followed by 30 cycles of 94°C for 30s, 55°C for 30 s, 72°C for 1 min, ended with an extension step of 72°C for 7 min. Two allele-specific amplicons and a common outer amplicon could be amplified by using four primers CF, CR, TF, TR (S3 Table). The PCR amplicons were resolved by using electrophoresis on a 1.8% agarose gel.

## Results

### Characterization of the mutant *ts1*

**Fig 1:**
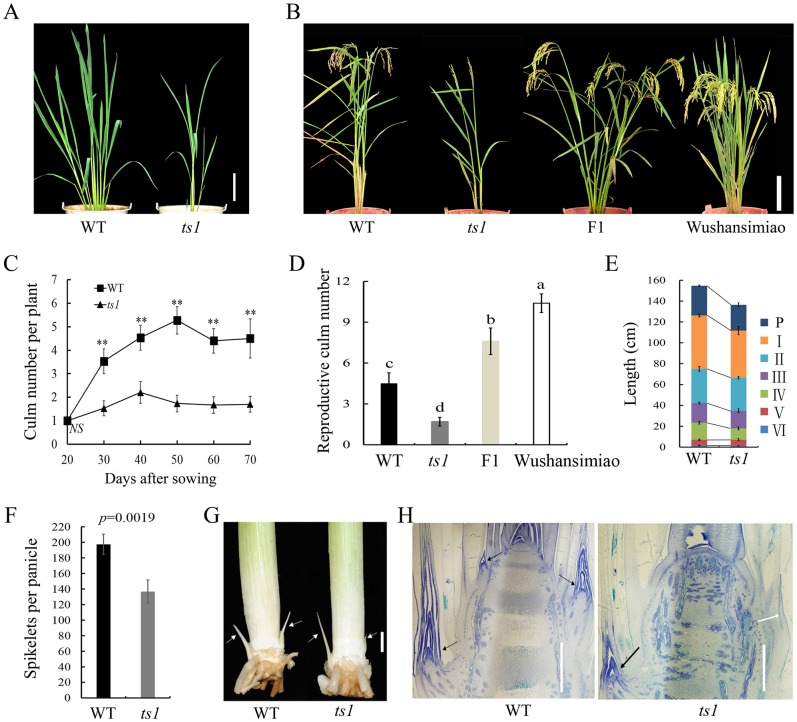
Phenotypes of the *ts1* mutant and its wild type. (A) Plant architecture of the *ts1* mutant and its wild type at tillering stage. Bar = 20 cm. (B) Plant architecture of wild type, the *ts1* mutant, *ts1*/wushansimiao F1 plants, and cv.Wushansimiao at maturity. Bar = 30 cm. (C) Comparison of culm numbers between the *ts1* mutant and its wild type. (D) Comparison of panicle numbers among LY95, *ts1*, *ts1*/Wushansimiao F1 plant, and Wushansimiao. (E) Comparison of panicle length and internode lengths between the *ts1* mutant and its wild type. P represents panicle length; I, II, III, IV, V, VI represent the first, second, third, fourth, fifth, sixth internode length, respectively. (F) Comparison of spikelets per panicle between the *ts1* mutant and its wild type. (G) Morphological observation of tiller buds of the *ts1* mutant and its wild type. White arrows point to the position of tiller buds. Bar = 3 mm. (H) Longitudinal sections of shoot apexes of the *ts1* mutant and its wild type. Black arrows indicate active tiller buds, white arrow indicates inactive tiller buds. Bar = 200μm. WT, wild type rice line LY95. Each value represents the mean ± SE of 10 replicates. Significant differences of mean values in (C), (E), and (F) were determined by the Student’s *t*-test. Double asterisks indicate significant differences at *P*<0.01. Significant differences of mean values in (D) were determined by the Duncan test. a, b, c, and d represent 4 levels of significant differences at *P*<0.05.

Through the utilization of the chemical mutagen EMS, we isolated a rice tiller suppression mutant *ts1* from its wild type line LY95 (Fig 1). In field conditions, *ts1* produced only 1.53 ± 0.32 and 2.20 ± 0.47 culms (one main culm with its tillers) while LY95 generated 3.53 ± 0.53 and 4.53 ± 0.53 culms at 30 and 40 DAS, respectively (Fig 1A and 1C). At maturity, *ts1* had only 1.70 ± 0.34 reproductive culms per plant while LY95 harvested 4.50 ± 0.83 reproductive culms per plant (Fig 1B and 1D). Among the observed *ts1* plants, 80% of them produced no more than one tiller during the whole growing season, and the other 20% plants could develop only two tillers at the tillering peak stage (40 DAS) (Fig 1A and 1C). Meanwhile, we sampled the tiller buds of the representative wild type and *ts1* plants for further morphological and histological studies. The results revealed that only one tiller bud of *ts1* has normal morphological and histological status as compares with the wild type tiller buds, while the other tiller bud of *ts1* was much smaller (Fig 1G) and had no meristem to be observed (Fig 1H). Therefore, the tiller suppression phenotype of *ts1* plants could probably be attributed to the abnormal formation of their tiller buds at tillering stage. The gene responsible for the tiller suppression phenotype in the mutant *ts1* was therefore designated as *tiller suppression 1* (*ts1*).

Besides, the *ts1* mutant has a shorter plant height and panicles with less spikelets (Fig 1E and 1F). At maturity, plant height of *ts1* (136.52 ± 2.47 cm) was decreased by 11.77% as compared with its wild type (154.73 ± 1.96 cm) (Fig 1E). Panicle length of *ts1* was about 3.7 cm shorter (24.77 ± 1.75 cm) than wild type (28.47 ± 0.73 cm) (Fig 1E). We further dissected plant height trait by analyzing internode elongation pattern of both *ts1* and its wild type. Among the six internodes, the first and forth internodes in the *ts1* mutant were deceased by 11.82% and 33.48% in length, respectively (Fig 1E), which resulted in the dwarf phenotype of *ts1*. Spikelsts per panicle of *ts1* (136.90 ± 14.87) was about 30.75% less than its wild type (197.70 ± 12.90) (Fig 1F).

To investigate the inheritance pattern of the *ts1* mutant, we investigated culm number of the *ts1*/Wushansimiao F1 plants and both of their parents at maturity. The *ts1* plants had 1.7 ± 0.34 culms, while its F1 plants had 7.6 ± 1.96 culms per plant, a value significantly biased towards the wild type parent Wushansimiao (9.75 ± 1.35 culms) (Fig 1B and 1F). By Formula (1), dominance degree was calculated as -0.47, indicating that as for the trait culm number, *ts1* was an incompletely recessive mutant relative to the wild type parent Wushansimiao.

### Genetic analysis and QTL mapping of the target *ts1* locus

**Fig 2:**
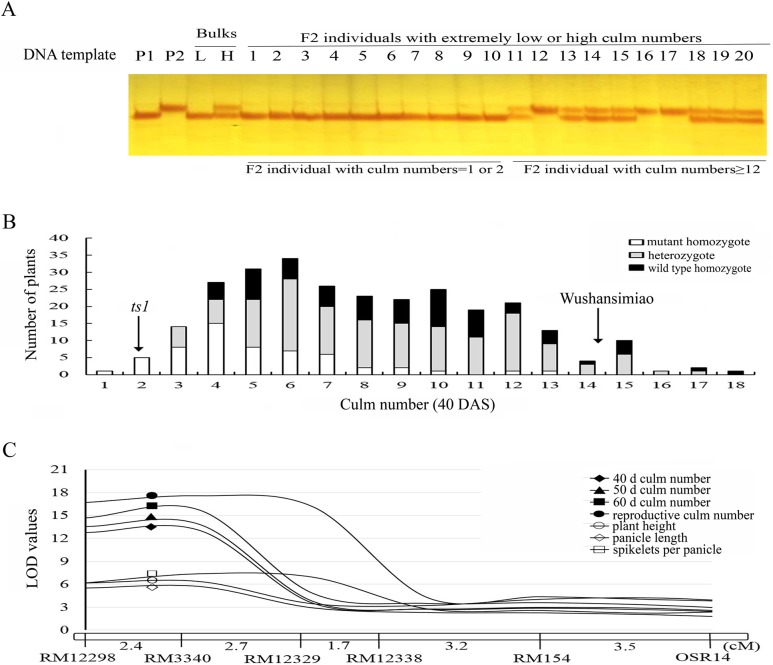
Primary mapping and QTL analysis of *ts1* locus in *ts1*/Wushansimiao F2 population. (A) Genotypes of SSR marker RM3340 for a pair of bulks and F2 individuals with extreme trait values. P1, *ts1*; P2, cv.Wushansimiao; L, L bulk; H, H bulk; Lanes 1–10 are F2 individuals with culm numbers = 1 or 2. Lane 11–20 are F2 those with culm numbers ≥12 at 40 DAS. Culm number of *ts1* (P1) was 2.2 while Wushansimiao (P2) was 14.5 (40 DAS). (B) Frequency distribution of culm number at 40 DAS in *ts1*/Wushansimiao F2 population and these individual’s genotypes for marker RM3340. (C) LOD score plots showing locations of QTL for culm numbers at 40d, 50d and 60d, reproductive culm number, plant height, panicle length, and spikelets of main panicle within RM12298-RM154 on the short arm of chromosome 2. LOD curve indicates the strength of evidence for the presence of the QTL. Marker names and genetic distances between markers are shown below the chromosome.

In the *ts1*/Wushansimiao F2 population, the trait culm number (at 40DAS) showed a continuous distribution and approximately 92% of the F2 individuals had trait values between both parents (Fig 2B). In order to genetically localize the gene *ts1* in the mutant, the bulked segregant analysis was conducted. A total of 269 polymorphic markers distributed across all of the 12 rice chromosomes were used to detect difference between the L (low tilering) and H (high tillering) bulks. Among those markers, two linked ones RM3340 and RM154 were found to differentiate the two bulks. For these two markers, the L bulk and all of the F2 individuals that constituted the L bulk showed a genotype the same as the mutant *ts1*, while the H bulk had a heterozygous genotype. The F2 individuals that constituted the H bulk showed a genotype the same as the wild type LY95 or a heterozygous genotype (Fig 2A). These results suggested that the mutant-carried tiller suppression gene *ts1* was putatively linked to markers RM3340 and RM154 that located on the short arm of chromosome 2.

**Fig 3:**
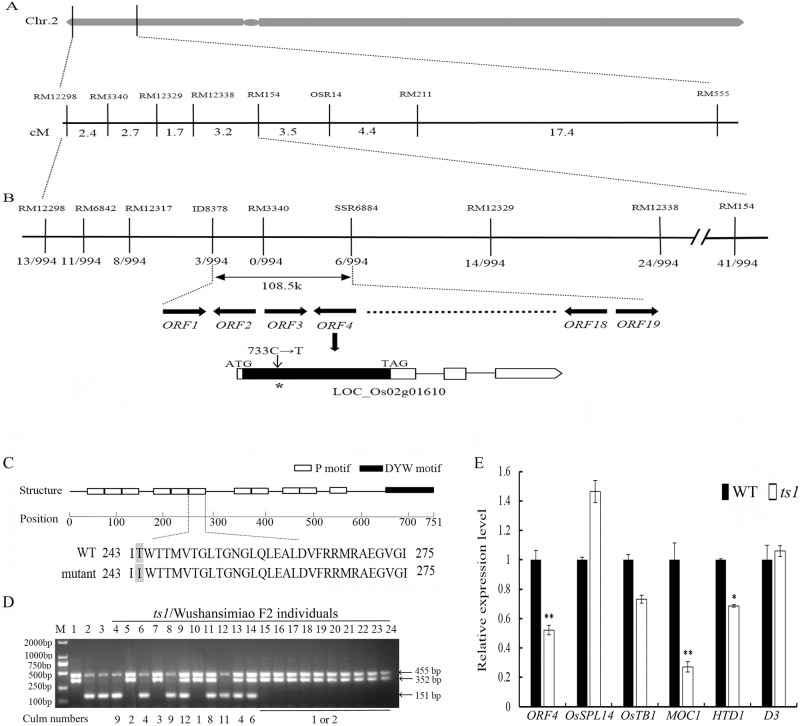
Fine mapping and candidate analysis of *ts1*. (A) Genetic map around the *ts1*-linked two markers RM3340 and RM154 on the short arm of rice chromosome 2. (B) Fine mapping of the *ts1* locus. The *ts1* locus was narrowed down to a 108.5 kb region between ID8378 and SSR6884 with nineteen predicted *ORFs*. Numbers below the chromosome bar were recombinant frequency. Mutant allele of *ORF4* contains a c.+733C→T point mutation when compared with its wild allele. (C) Structure and sequence analysis of the predicted *ORF4* protein. At +244 position in the sixth P subfamily motif of *ORF4*, the mutant protein harbored a threonine to isoleucine substitution. Amino acid with grey background indicates the point substitution position, amino acids without colorful background were consensus amino acids. White rectangle represents P motif, black rectangle represents DYW motif. (D) PCR-based SNP assay of 24 representative rice materials by SNP maker cd-733C/T. M, 2000 bp DNA marker; 1, *ts1*; 2, LY95; 3, cv.Wushansimiao; 4–14, randomly selected *ts1*/Wushansimiao F2 individuals; 15–24, segregants from recessive-class population for fine mapping. Numbers below PCR amplicons were culm numbers of each F2 individual. (E) Expression of the candidate gene *ORF4* and other five rice tillering regulation genes *OsSPL14*, *OsTB1*, *MOC1*, *HTD1*, *D3* in the *ts1* mutant. Relative expression levels in the *ts1* mutant and its wild type were detected by quantitative RT-PCR, shown as mean ±SE of three separate experiments. * and ** indicate significances at *p*<0.05 and *p*<0.01 by the Student’s *t*-test, respectively.

To precisely determine the marker interval for the *ts1* locus, 279 F2 individuals were randomly selected from the *ts1*/Wushansimiao F2 population and then genotyped with molecular markers around RM3340 and RM154 on the short arm of rice chromosome 2 (Figs 2C and 3A). A local linkage map around the putatively linked markers RM3340 and RM154 were constructed (Fig 2C). Culm numbers at 40, 50, 60 DAS and maturity for each of the 279 F2 individuals were investigated for QTL analysis. The results indicated that there was indeed a major QTL localized in marker interval RM12298-RM154. It has a major effect on culm number, explaining 19.5–25.3% of the trait variance with a high LOD score of 13.1–17.6. The QTL also showed a pleiotropic effect on other traits viz. plant height, panicle length and spikelets per panicle, explaining 8.2–13.4% of trait variances with LOD values of 5.7–7.4 (Fig 2C).

### Fine mapping of the target gene *ts1*

Given that *ts1* was an incompletely recessive mutant to Wushansimiao, the recessive-class analysis was applied to fine map the gene *ts1*. From a large *ts1*/Wushansimiao F2 population with 9700 individuals, we picked out a total of 497 F2 individuals with one or two culms to construct a fine mapping population. For these individuals, their recessive phenotypes were further confirmed by their F3 progeny test. All of the 497 individuals were genotyped with target region-flanking SSR markers RM12298 and RM154. As a result, 13 and 41 recombinant events were identified with RM12298 and RM154, respectively (Fig 3B). Subsequently, 7 polymorphic markers were developed to construct a high resolution genetic map covering the *ts1* locus (S1 Table). For these markers RM6842, RM12317, ID8378, RM3340, SSR6884, RM12329 and RM12338, there were 11, 8, 3, 0, 6, 14 and 24 recombinant events to be identified, respectively (Fig 3B). RM3340 was found to co-segregate with the *ts1* phenotype, and the *ts1* locus was precisely defined to a 108.5 kb region between markers ID8378 and SSR6884.

By using the cosegregated marker RM3340 (*r* = 0), we analyzed the genetic model of the gene *ts1* in the *ts1*/Wushansimiao F2 population. Among the 279 individuals randomly sampled from the *ts1*/Wushansimiao F2 population, 57 mutant homozygotes, 149 heterozygotes and 73 wild type homozygotes were detected with marker RM3340, respectively. Their corresponding average values in trait culm number at 40 DAS were 5.05 ± 2.39, 8.68 ± 3.28 and 9.00 ±3.31, respectively (Fig 2B). By Formula (2) described above, dominance degree of the target gene *ts1* was calculated as -0.79, indicating that the *ts1* allele was incompletely recessive to its wild type allele *TS1* in Wushansimiao.

### Candidate gene analysis for *ts1*

According to the MSU Rice Genome Annotation Project Database (http://rice.plantbiology.msu.edu/index.Shtml) and the RiceGAAS (http://ricegaas.dna.affrc.go.jp), there are nineteen *ORFs* predicted in the *ts1* locus-contained 108.5 kb genomic region of cv. Nipponbare. Among them, eight genes have been predicted to be functional and the others included two expressed proteins without annotation, two transposon proteins and seven retrotransposon proteins (Table 1). None of the nineteen *ORFs* have been previously reported to be involved in rice tillering. Therefore, *ts1* might be a novel gene responsible for rice tillering. Based on the predicted gene annotations and the physical location (three recombinant events were detected with the left flanking marker ID8378 and six with the right flanking marker SSR6884), seven functional genes (*ORF3*, *ORF4*, *ORF12*, *ORF13*, *ORF14*, *ORF15*, *ORF16*) in the candidate region were selected for genomic DNA sequencing with a PCR approach (Table 1 and S2 Table). As a result, only one genomic sequence polymorphism was found in *ORF4* (LOC_Os02g01610). In the only one exon of *ORF4*, the mutant allele harbored a C to T substitution at +733 site as compared with its wild allele (Fig 3B). Such a substitution led to a threonine to isoleucine substitution in the amino acid sequence of the mutant protein. We also analyzed the structure of the mutant protein with an online protein structure analysis tool: SMART (http://smart.embl-heidelberg.de/). The result revealed that the protein is a PPR type with eleven P motifs and a DYW motif tail. The threonine to isoleucine substitution was precisely occurred at +244 position in the sixth P motif of the mutant protein (Fig 3C).

**Table 1:** Candidate genes in the genomic region between ID8378 and SSR6884.

*ORF*	Gene ID	Putative function	Sequencing
*ORF1*	LOC_Os02g01570	Retrotransposon protein, putative	-
*ORF2*	LOC_Os02g01580	Retrotransposon protein, putative	-
*ORF3*	LOC_Os02g01590	Glycosyl hydrolases, putative, expressed	Identical
*ORF4*	LOC_Os02g01610	Pentatricopeptide, putative, expressed	C to T substitution
*ORF5*	LOC_Os02g01620	Transposon protein, putative	-
*ORF6*	LOC_Os02g01630	Retrotransposon protein, putative	-
*ORF7*	LOC_Os02g01640	Retrotransposon protein, putative	-
*ORF8*	LOC_Os02g01660	Retrotransposon protein, putative	-
*ORF9*	LOC_Os02g01670	Retrotransposon protein, putative	-
*ORF10*	LOC_Os02g01679	Retrotransposon protein, putative	-
*ORF11*	LOC_Os02g01690	Transposon protein, putative	-
*ORF12*	LOC_Os02g01700	RNA recognition motif containing, putative, expressed	Identical
*ORF13*	LOC_Os02g01710	Peptidase family C78 domain containing protein, expressed	Identical
*ORF14*	LOC_Os02g01720	Transmembrane protein 120A, putative, expressed	Identical
*ORF15*	LOC_Os02g01730	Serine/threonine-protein kinase At1g18390 precursor, putative, expressed	Identical
*ORF16*	LOC_Os02g01740	U5 small nuclear ribonucleoprotein 200kDa helicase, putative, expressed	Identical
*ORF17*	LOC_Os02g01750	Expressed protein	-
*ORF18*	LOC_Os02g01760	Diphosphomevalonate decarboxylase family protein, expressed	-
*ORF19*	LOC_Os02g01765	Expressed protein	-

We subsequently conducted quantitative RT-PCR analysis with samples of shoot apexes at tillering stage (40 DAS) and found that the relative expression level of *ORF4* in *ts1* plants significantly decreased by 47.8% in contrast to its wild type (Fig 3E). To investigate the relationship between *ts1* and other rice tillering regulators, gene expression analysis of other five rice tillering-related genes (*OsSPL14*, *OsTB1*, *MOC1*, *HTD1*, and *D3*) was performed. As compared with wild type plants, expression of *MOC1* and *HTD1* in the *ts1* mutant was significantly decreased by 77.30% and 31.39%, respectively. Expression levels of *OsSPL14*, *OsTB1*, and *D3* did not show significant difference between the *ts1* mutant and its wild type line. These results indicated that *ts1* may regulate rice tillering through *MOC1* and *HTD1* associated pathways.

### Development of a codominant SNP marker for *ts1*

Based on the c.+ 733C→T substitution in the mutant allele of *ORF4*, we developed a co-dominant SNP marker (cd-733C/T) by the tetra-primer ARMS-PCR method. Here, primer pair CF/CR produced a 151bp amplicon that represented the mutant allele while primer pair TF/TR generated a 352bp band that was specific to the wild type allele. Meanwhile, primer pair TF/CF produced a 455bp band that was common to both of the alleles. Therefore, the mutant homozygote could show a 151bp and a 455bp amplicons while the wild type homozygote could produce a 352bp and a 455bp amplicons, and the heterozygote could have all of the three amplicons.

When the marker cd-733C/T was used to conduct a PCR-based SNP assay, it was found that the F2 individuals with culm numbers 0–3 amplified the mutant allele the same as *ts1* while the F2 ones with culm number 4 or more than 4 amplified the wild type allele the same as the wild parents LY95 and Wushansimiao, or a heterozygous genotype (Fig 3D). Additionally, when genotyped with the SNP marker cd-733C/T, all amplicons of the segregants from the fine mapping population belonged to the mutant type (Fig 3D), which further demonstrated that the SNP marker co-segregated with the extremely low tillering phenotype.

Furthermore, using the SNP marker cd-733C/T, we genotyped 276 modern rice varieties or breeding lines and found that they all belonged to the wild type, the same as that for LY95. The result suggested that the C to T substitution of *ORF4* occurred in the *ts1* mutant might be a much rare natural variation.

## Discussion

Rice tillering is a complex biological process with two main stages: the formation of an axillary bud at each leaf axil and its subsequent outgrowth [5]. In rice monoculm mutants *moc1* and *moc3*, their tiller buds are defective in formation, and thus their plants keep monoculm during the whole growing stage [5, 7]. In monoculm mutant *moc2*, its tiller bud formation was detected, but no extension or outgrowth of tillers was observed. The researchers speculated that the growth of tiller buds was inhibited or the tiller buds failed to enlarge to generate individual organ. Additionally, the researchers found that under high-temperature condition, the *moc2* mutant sometimes could produce one tiller [6]. In this study, 80% of the *ts1* plants kept monoculm or produced one tiller and the other 20% of them could produce only two tillers at tillering peak stage (40DAS). From the morphological study, we have observed one normally formed tiller bud in the *ts1* mutant, but the other tiller bud was abnormally formed because of no meristem (Fig 1H). Therefore, the tiller suppression phenotype of *ts1* could be probably attributed to the abnormal formation of most of their tiller buds. To a large extent, its morphological development features could be similar to that of the *moc* mutants as described above.

In rice, tillering has been found to be quantitative in nature [14]. Tiller number trait tends to show a continuous distribution in a segregated population, which indicates that multiple genes or QTLs could be involved in its regulation. In order to fine map the genes for trait tiller number, researchers usually have to develop advanced segregating populations such as near-isogenic lines (NILs) or chromosome segments substitution lines (CSSLs), so as to avoid genetic background noise. With this strategy, the genes *Ltn* for low tiller number and *qpn1* for decreased panicle number per plant in rice have been fine mapped to a 38.6 kb-region on the long arm of chromosome 8 and a 34.4 kb region on the long arm of chromosome 1, respectively [26, 27]. In spite of these, however, mapping of *dit1* appears to suggest that to develop advanced segregating populations such as NILs or CSSLs is not always prerequisite for fine mapping of the genes underlying such a quantitative trait. In the mapping case of the EMS-induced *dit1* allele for dwarf and increased tillering, the gene on chromosome 4 could explain 19.79% of tiller number variation in its F2 mapping population [14]. In this study, trait culm number was also found to show a continuous distribution in the *ts1*/Wushansimiao F2 population. The BSA and QTL mapping indicated a major gene localized in interval ID8378-SSR6884 of chromosome 2 and explained 19.5%-25.3% of the total culm number variation. With such a F2 population, the target gene (*ts1*) has been successfully fine mapped to a 108.5 kb region on chromosome 2 by the subsequent RCA. Taking the cases of both *dit1* and *ts1* into consideration, we attribute their success to two factors. One is the target gene explaining as much as about 20% or more of the trait variation in its population, which could make the BSA to be more effective in identifying markers putatively linked to the target gene. The second is the selection of extreme individuals out of the population and the validation of their recessive genotypes by their progenies. This step could be crucial in avoiding genetic background noise for the use of the RCA method. The current study suggested that, if the above two conditions were met, the combination strategy of both the BSA and the RCA could be a powerful and time-saving way in mapping of genes for the target trait even though the trait showed a somehow continuous variation in a population.

So far, a number of tillering genes or QTLs have been genetically mapped (http://archive.gramene.org/qtl/). Among them, only two QTLs for tillers per plant were mapped on the short arm of chromosome 2 [28, 29]. Up to now, neither of them has been reported to be fine mapped. Besides, according to their physical positions, both of them had no overlapping region with the interval ID8378-SSR6884 for *ts1* identified in this study. Therefore, *ts1* could be a novel gene for rice tillering.

Keeping in view the results of candidate gene analysis and SNP assay, *ORF4* (LOC_Os02g01610), encoding a PPR protein, was identified as a strong candidate gene for the *ts1*. Previous studies on rice PPR proteins demonstrated that they are RNA-binding proteins. A typical PPR protein binds one or several organellar transcripts and influences their expression by altering RNA sequence, turnover, processing, or translation [30]. Mutation harbored in the P motif could usually lead to altered gene expression level. For example, a rice PPR protein named wsl has two amino acid deletion in its third P motif which resulted in aberrant transcript accumulation and its product reduction in the mutant [31]. In this study, as a strong candidate gene for the *ts1*, LOC_Os02g01610 was found to have a point mutation in the sixth P motif of the *ts1* mutant protein and the mutation might result in its altered expression and then in its mutant phenotype. Of course, the suggestion remains to be clarified by next genetic complement experiments.

The molecular mechanisms for rice tillering have been illustrated in a number of reports, such as the *MOC1* asscociated pathway [5], the strigolactones pathway [2], and the *OsTB1* associated pathway [17], in which a lot of characterized genes were involved. The current study chose five tillering regulated genes and detected their relative expression level in the *ts1* mutant. It was found that the relative expression levels of *MOC1* and *HTD1* in the *ts1* mutant were significantly lower than that in wild type plants. *MOC1* is known to be expressed mainly in axillary buds and it functions to initiate axillary buds and promote their outgrowth [5]. Therefore, it was speculated that the down-regulated *MOC1* in the *ts1* mutant could contribute to the abnormal initiation and formation of the *ts1* tiller buds and finally lead to the tiller suppression phenotype. *HTD1* was reported to mainly express at stem node where axillary meristems initiate and its defect can lead to dwarf plant height and high tillering [15]. In this study, the down-regulated *HTD1* in the *ts1* mutant might be one of the reasons for the shorten plant height of the *ts1* plants. In general, the above results will provide new clues for us to understand the molecular basis of rice tillering regulation.

To date, applications of MAS in rice plant type improvement are still limited. In this study, based on the c.+ 733C→T substitution found in *ts1* candidate gene *ORF4* (LOC_Os02g01610), we developed a co-dominant SNP marker cd-733C/T by using the tetra-primer ARMS-PCR method. SNP assay proved it co-segregated with the tiller suppression phenotype. Due to its being genetically co-dominant and specific to the gene *ts1*, this SNP marker could be useful in marker-assisted selection of *ts1* allele in rice plant architecture improvement programs.

## Supporting Information

S1 Table PCR primer sequences for gene fine mapping. (DOCX)

S2 Table PCR primer sequences for ORF sequencing. (DOCX)

S3 Table SNP marker and Q-RT-PCR primer sequences. (DOCX)

## Abbreviations

ARMS-PCR: amplification refractory mutation system polymerase chain reaction
BSA: bulked segregant analysis
cM: centi Morgan
CTAB: hexadecyl trimethyl ammonium bromide
CSSLs: chromosome segment substitution lines
DAS: days after sowing
EMS: ethyl methylsulfonate
FAA: formalin/acetic acid/alcohol
InDel: insertion deletion
LOD: logarithm of odds
NILs: near isogenic lines
NPT: near plant type
ORFs: open reading frames
PPR: pentatricopeptide repeat
QTL: quantitative trait locus
Q-RT-PCR: quantitative—real time–polymerase chain reaction
SNP: single nucleotide polymorphism
SSR: single sequence repeats
