# Supplementary material for: Fine Mapping and Candidate Gene Analysis of the Tiller Suppression Gene ts1 in Rice
Source: PLoS One. 2017 Jan 20;12(1):e0170574. doi: 10.1371/journal.pone.0170574 (PMC5249193; doi:10.1371/journal.pone.0170574)
Supplement: S1 Table — (DOCX) [file pone.0170574.s001.docx]

| Primers | Forward primer | Reverse primer |
| --- | --- | --- |
| RM12298 | GGGTGCGGTACTTAGCTACTCC | CTGTCTACTTGCATTGCTATCTCC |
| RM6842 | CCGTGCATCTCGCTACCTAACC | TGCACACACAACTTAGAGGAAGAAGG |
| RM12317 | ATAATTGTGGTCCCGAGTCATCC | TGCTTCTTCAACTCAACTCTCAGC |
| ID8378 | GGAATCTAGACACCAGAACC | TCAATGCTAGTTTGAGGGTT |
| RM3340 | GAGAGAGACACCAAATGATCCATCC | ACTGATTTGGCCCTTGTTCTTGG |
| SSR6884 | CGGATGTGATGTGAGCAA | CACCTGCAACAGATATTCCAAA |
| RM12329 | AGGAAGAGGCGAAGGTAGATCG | CCAATCATGCTGTGTTTCAAGG |
| RM12338 | AGCTCAAGCTCAAGCTCACAACC | TGCACTGCAACCTAAACCTTTCC |
| RM154 | GACGGTGACGCACTTTATGAACC | CGATCTGCGAGAAACCCTCTCC |
