# Supplementary material for: Fine Mapping and Candidate Gene Analysis of the Tiller Suppression Gene ts1 in Rice
Source: PLoS One. 2017 Jan 20;12(1):e0170574. doi: 10.1371/journal.pone.0170574 (PMC5249193; doi:10.1371/journal.pone.0170574)
Supplement: S2 Table — (DOCX) [file pone.0170574.s002.docx]

| Primers | Forward primer | Reverse primer |
| --- | --- | --- |
| ORF3-Seq1 | ATACATCCTCCGACCCCTCG | GAAGAACGAACGAACAGAGCG |
| ORF3-Seq2 | CCCGAGAGGAACTGGATGAAC | CAATGGAGCCCAGTAAAAGTGC |
| ORF3-Seq3 | TGGGCTCCATTGACACGTTG | GGCATTTAACAGTTCCAGCGG |
| ORF4-Seq1 | GGCAAATCTGGAACGCTAGG | CGGGTATGTGTGGATTGTCAC |
| ORF4-Seq2 | CGAGCACGTGCCATCACTAA | CGCGGGCACTTCTAGTCT |
| ORF12-Seq1 | TAGGCGTAGGCTAAGCACAACC | CAGGGGAGTGGTGAAACATCT |
| ORF12-Seq2 | GATGTTTCACCACTCCCCTGT | CTAGTCTACTCGAGAACGCCAC |
| ORF13-Seq1 | ACGTCTCCGATTACCAACCAG | GCATCCCAAGCCTTGTAGTC |
| ORF14-Seq1 | GTGAGCTGAAAGCATGGCAG | TGTTGGCGACTGAGATCCTG |
| ORF14-Seq2 | GAAACATTGTCCACTGCTCCG | GGACCGGGTAAAGTAGCAAGA |
| ORF15-Seq1 | AACAGTCCACAGCTCAGACC | CCTCGTCTCGGTGTGGTTAAA |
| ORF15-Seq2 | TTTAACCACACCGAGACGAGG | GAACCCATTTTCTGCTCTGCTC |
| ORF16-Seq1 | AGGGCTACAACTTGATGGGC | CGATCTGGTCGAACATCTGGT |
