# Supplementary material for: Fine Mapping and Candidate Gene Analysis of the Tiller Suppression Gene ts1 in Rice
Source: PLoS One. 2017 Jan 20;12(1):e0170574. doi: 10.1371/journal.pone.0170574 (PMC5249193; doi:10.1371/journal.pone.0170574)
Supplement: S3 Table — (DOCX) [file pone.0170574.s003.docx]

| Primers | Forward primer | Reverse primer | Use of Primers |
| --- | --- | --- | --- |
|  |  |  | **SNP assay** |
| C allele primers^a^ | TGTTTCAAGAAATGGAGGC | AGTCCTGTAACCATCGTAGTCCTAG^b^ |  |
| T allele primers^a^ | ATATCCAACAATCATCGCAGTC | GATGGTGGACAGGGATTCCATAAT^b^ |  |
| Outer primers^a^ | ATATCCAACAATCATCGCAGTC | TGTTTCAAGAAATGGAGGC |  |
|  |  |  | **Q-RT-PCR** |
| *ORF4*-Q | AGCTGATGGTGGACAGGGAT | ATCAAGAGCCTCCAACTGCA |  |
| *OsSPL14-*Q | GGATATGGTGCCAACACATACAG | GACATGGCTGCAGCCTGGTTGTG |  |
| *OsTB1-*Q | TCCCATCAGTAAAGCATGCTTCC | GATGGTGGTGGTGGTGGTG |  |
| *MOC1-*Q | GCGTCATGTTCTTGCACAACC | CGAGTAGTGATCCATGGCCAC |  |
| *HTD1-*Q | GCTGCACATGTCAAGCTGCTC | TTCATGAACGACGGGTCCAG |  |
| *D3-*Q | GGATGGTGCCATTGCTCCAC | CGATAATGACATGCAAAACTGGC |  |
| *OsUBI*-Q | AACCAGCTGAGGCCCAAGA | ACGATTGATTTAACCAGTCCATGA |  |

a Put four primers into one PCR procedure simultaneously to distinguish mutant type, wild type, and heterozygous genotypes.

b Underline shows position of an artificial base pair mismatch
